# Carotid Free-Floating Thrombus: A Case Series, Narrative Review, and Proposal for Biology-Guided Treatment Selection

**DOI:** 10.3390/medsci14040457

**Published:** 2026-08-06

**Authors:** Spyros Papadoulas, Kate Tabaku, Chrysanthi Papageorgopoulou, Konstantinos Nikolakopoulos, Zafeiria Papathanassiou, Helen Kourea, Petros Zampakis, Vasilios Panagiotopoulos, John Ellul, Francesk Mulita, Vasileios Leivaditis

**Affiliations:** 1 Department of Vascular Surgery, General University Hospital of Patras, Patras Medical School, 26504 Patras, Greece; katetabaku@gmail.com (K.T.); chrisanthi.papageorg@gmail.com (C.P.); konstantinosn@yahoo.com (K.N.); 2 Department of Radiology, General University Hospital of Patras, 26504 Patras, Greece; papaze74@gmail.com (Z.P.); pzampakis@gmail.com (P.Z.); 3 Department of Pathology, General University Hospital of Patras, 26504 Patras, Greece; hkourea@yahoo.com; 4 Department of Neurosurgery, General University Hospital of Patras, 26504 Patras, Greece; panagiotopoulos2000@yahoo.com; 5 Department of Neurology, General University Hospital of Patras, 26504 Patras, Greece; ellul@upatras.gr; 6 Second Department of Surgery, Medical School, Democritus University of Thrace, 68100 Alexandroupolis, Greece; 7 Department of Cardiothoracic and Vascular Surgery, Westpfalz Klinikum, 67655 Kaiserslautern, Germany; vnleivaditis@gmail.com

**Keywords:** carotid free-floating thrombus, ischemic stroke, carotid endarterectomy, anticoagulation, endovascular thrombectomy, optical coherence tomography, precision medicine

## Abstract

*Background:* Carotid free-floating thrombus (CFFT) is an uncommon but clinically significant cause of transient ischemic attack and ischemic stroke, carrying a substantial risk of recurrent cerebral embolization. Despite advances in vascular imaging, the optimal management of CFFT remains controversial because available recommendations are largely based on retrospective studies, case series, and expert opinion. The present study reports a single-center experience with CFFT and provides an updated narrative review of current diagnostic and therapeutic strategies, with particular attention to the timing of intervention. *Methods:* A retrospective review of patients diagnosed with CFFT and managed at our institution over a 20-year period was performed. Clinical presentation, imaging findings, treatment strategy, and outcomes were analyzed. In parallel, a narrative review of the contemporary literature was conducted to summarize current evidence regarding medical management, carotid endarterectomy, endovascular techniques, hybrid approaches, and emerging concepts related to thrombus composition and maturation. *Results:* Five patients with symptomatic CFFT were identified. Four patients underwent carotid thromboendarterectomy, whereas one patient was managed medically with anticoagulation and antiplatelet therapy, resulting in complete thrombus resolution. Two patients experienced neurological deterioration while receiving initial anticoagulation and subsequently required urgent surgical intervention. No postoperative strokes occurred after carotid endarterectomy. Review of the literature confirmed that anticoagulation remains the cornerstone of initial therapy and achieves thrombus resolution in a substantial proportion of patients. However, recurrent neurological events continue to occur under medical treatment, while the indications and timing of invasive intervention remain poorly defined. Recent advances in high-resolution magnetic resonance imaging and optical coherence tomography suggest that thrombus age and composition may become important determinants of future treatment selection. *Conclusions:* CFFT represents a rare but potentially unstable vascular condition for which high-quality evidence is still lacking. Initial anticoagulation remains the most widely accepted treatment strategy, while carotid endarterectomy, endovascular thrombectomy, carotid artery stenting, and hybrid procedures may be appropriate in selected patients. Future management algorithms may ultimately move beyond a one-size-fits-all approach by incorporating thrombus biology, imaging characteristics, and individual patient risk profiles. Although this concept remains preliminary, advances in thrombus characterization may provide the foundation for more personalized treatment strategies as further clinical evidence becomes available.

## 1. Introduction

**Figure 1:**
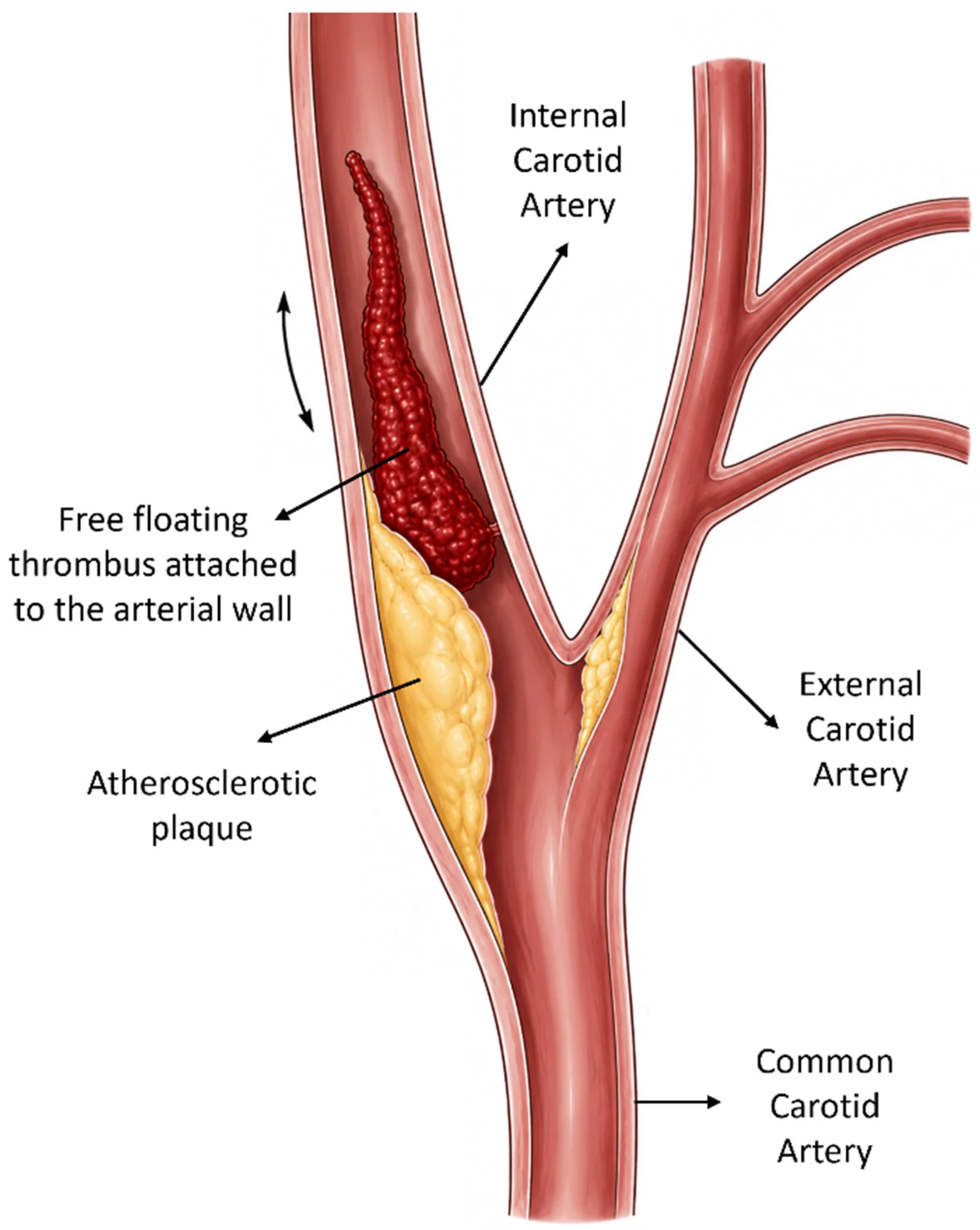
Schematic representation of a carotid free-floating thrombus (CFFT) originating from an atherosclerotic plaque at the carotid bifurcation and extending into the lumen of the internal carotid artery.

Carotid free-floating thrombus (CFFT) is defined as an elongated thrombus attached to the arterial wall, with circumferential blood flow detectable around its distal portion and mobility influenced by the pulsatile cardiac cycle (Figure 1). Although uncommon, CFFT represents a clinically important cause of transient ischemic attack (TIA) and ischemic stroke, accounting for approximately 1.3% of stroke presentations [1]. Its significance lies not only in the initial cerebrovascular event but also in the considerable risk of recurrent embolization and neurological deterioration during the early phase of the disease.

The diagnosis of CFFT has become increasingly straightforward with modern vascular imaging. Computed tomography angiography (CTA), in particular, has emerged as the diagnostic modality of choice because of its wide availability, rapid acquisition, and high diagnostic accuracy [2]. Nevertheless, despite improvements in diagnostic capabilities, considerable uncertainty remains regarding the optimal management of these patients. Current treatment recommendations are largely derived from retrospective studies, case reports, and small case series, reflecting the rarity of the condition and the absence of high-quality prospective evidence. Consequently, the 2023 European Society for Vascular Surgery (ESVS) guidelines provide only a weak recommendation (Class IIb) regarding invasive treatment strategies [3]. Similarly, the most recent guidelines from the Society for Vascular Surgery (SVS), the American Heart Association (AHA), and the European Stroke Organisation (ESO) do not provide specific recommendations for the management of symptomatic CFFT [4].

Although recent systematic reviews have substantially improved the understanding of CFFT management, important uncertainties remain regarding patient selection, procedural timing, and the potential role of thrombus biology in individualized treatment decisions.

In the present study, we report our single-center experience with CFFT over a 20-year period and provide a focused narrative review of the contemporary literature. Rather than attempting another comprehensive review of CFFT diagnosis and management, which has recently been addressed in several high-quality systematic reviews, our primary objective is to integrate current evidence with emerging concepts of thrombus biology. Specifically, we discuss how thrombus maturation, histopathological composition, and advanced imaging modalities may contribute to a more individualized, biology-guided approach to treatment selection and procedural timing. This conceptual framework is intended to complement, rather than replace, existing management strategies and to highlight promising directions for future research.

## 2. Materials and Methods

This study combines a retrospective case series with a narrative review of the contemporary literature on CFFT.

For the case series, all patients diagnosed with CFFT and managed at our institution over a 20-year period were retrospectively identified from departmental records. Clinical presentation, imaging findings, anatomical location of the thrombus, treatment strategy, timing of intervention, perioperative course, and clinical outcomes were reviewed. The diagnosis of CFFT was established using vascular imaging modalities, including color duplex ultrasonography and CTA. Additional imaging studies, such as magnetic resonance imaging (MRI) or magnetic resonance angiography (MRA), were obtained when clinically indicated. For the purposes of this study, CFFT was defined as an elongated intraluminal thrombus attached to the carotid arterial wall, with circumferential blood flow surrounding its distal free-floating portion, as demonstrated by color duplex ultrasonography and confirmed by CTA whenever available. The same radiological definition was applied retrospectively to all cases throughout the study period.

All consecutive patients diagnosed with CFFT and managed at our institution between 2005 and 2025 were retrospectively identified through a review of departmental clinical records, operative databases, and vascular imaging archives (color duplex ultrasonography and CTA). No eligible cases meeting the diagnostic criteria were excluded.

The therapeutic approach was individualized according to the patient’s neurological status, thrombus characteristics, extent of underlying carotid disease, and response to initial medical treatment. Both medical and surgical treatment strategies were analyzed. Follow-up imaging and clinical outcomes were reviewed whenever available.

In parallel, a narrative review of the literature was performed to provide an updated overview of the current understanding of CFFT. Relevant publications addressing epidemiology, pathophysiology, imaging, medical management, carotid endarterectomy, endovascular interventions, hybrid techniques, and treatment timing were identified through a search of the PubMed database. A literature search was performed in PubMed in March 2026 using combinations of the terms “carotid free-floating thrombus”, “floating thrombus”, “carotid thrombosis”, “carotid endarterectomy”, “anticoagulation”, “endovascular treatment”, “thrombectomy”, and “stroke”. Publications from database inception to March 2026 were considered. Clinical studies, systematic reviews, meta-analyses, guideline documents, and representative case series published in English were preferentially included according to their relevance to the objectives of this narrative review.

The objective of the review was not to perform a quantitative synthesis of the available evidence, but rather to summarize current treatment strategies, highlight areas of controversy, and discuss emerging concepts that may influence future management, including thrombus composition, thrombus maturation, and advanced imaging techniques.

## 3. Case Series

Between 2005 and 2025, five patients with symptomatic CFFT were managed at our institution (Table 1). All patients were male and presented with either transient ischemic attack or ischemic stroke. The thrombus was identified by vascular imaging and was most commonly associated with underlying atherosclerotic disease. Initial management included antithrombotic therapy in all cases; however, treatment strategies subsequently differed according to clinical presentation, thrombus characteristics, and response to medical therapy. Four patients ultimately underwent carotid thromboendarterectomy, whereas one patient was successfully managed with anticoagulation alone. The following cases illustrate the heterogeneity of CFFT presentation and highlight some of the challenges encountered in treatment selection and timing.

### 3.1. Case 1

A 35-year-old man presented with transient numbness of the right upper extremity, consistent with a transient ischemic attack (TIA). Color duplex ultrasonography and CTA revealed a free-floating thrombus at the left carotid bifurcation. Notably, no underlying vascular disease was known prior to presentation. Therapeutic anticoagulation with low-molecular-weight heparin (LMWH) was initiated.

Despite treatment, the patient developed Broca’s aphasia the following day, most likely as a result of recurrent cerebral embolization. Repeat vascular imaging was not performed because neurological deterioration prompted immediate surgical intervention. An urgent thrombectomy was therefore performed through a standard carotid endarterectomy (CEA) approach with primary closure. The postoperative course was uneventful, and no further neurological events occurred. However, the aphasia persisted, with only minimal improvement during long-term follow-up. The resulting communication impairment had a substantial impact on the patient’s quality of life and was ultimately associated with the development of depression.

### 3.2. Case 2

A 40-year-old man presented with right-sided hemiplegia. Color duplex ultrasonography and CTA demonstrated a free-floating thrombus at the left carotid bifurcation. Therapeutic anticoagulation with LMWH was initiated, and a slight neurological improvement was observed during the following days.

However, one week after presentation, the patient experienced neurological deterioration despite ongoing anticoagulation. Given the progression of symptoms, a carotid thromboendarterectomy was performed. No repeat imaging was obtained before surgery because the patient’s neurological deterioration necessitated urgent operative management. Intraoperatively, a small atherosclerotic plaque was identified as the likely substrate for thrombus formation. Interestingly, only a limited amount of residual thrombotic material was present at the time of surgery, suggesting partial thrombus resolution or distal embolization during the preceding days.

The postoperative course was uneventful, with no further neurological deterioration. Following recovery from surgery, the patient was transferred to a specialized rehabilitation center for continued neurological rehabilitation.

### 3.3. Case 3

A 60-year-old man presented with a TIA. Color duplex ultrasonography revealed a 70% stenosis of the left common carotid artery (CCA). CTA confirmed the stenotic lesion and demonstrated a superimposed free-floating thrombus extending approximately 1 cm within the CCA.

Therapeutic anticoagulation was initiated, and a carotid thromboendarterectomy with bovine patch angioplasty was performed the following day. The postoperative course was uneventful, and the patient was discharged on the second postoperative day without neurological deficits.

### 3.4. Case 4

**Figure 2:**
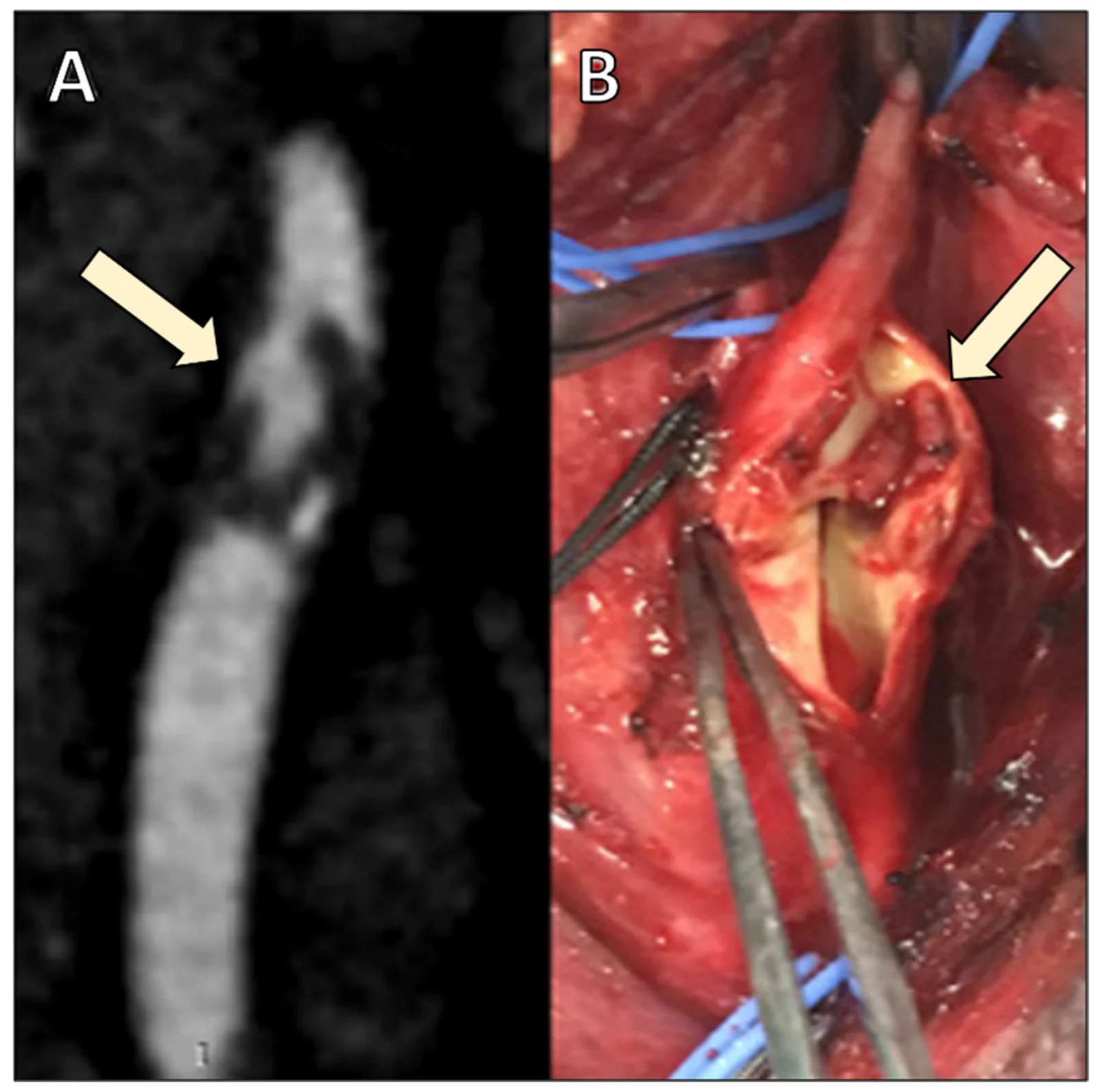
(**A**) Computed tomography angiography (CTA) demonstrating the characteristic “finger sign” of a carotid free-floating thrombus (CFFT) in the sagittal plane. (**B**) Intraoperative photograph obtained during carotid endarterectomy showing a free-floating thrombus arising from a small atherosclerotic plaque and extending into the lumen of the internal carotid artery.

A 58-year-old man presented with crescendo TIAs, characterized by recurrent episodes of right arm weakness and motor aphasia. Color duplex ultrasonography demonstrated an atherosclerotic plaque with an overlying thrombus, resulting in approximately 70% stenosis of the left internal carotid artery (LICA). CTA confirmed a high-grade stenosis of approximately 80% and revealed a free-floating thrombus at the carotid bifurcation (Figure 2).

Brain MRI demonstrated multiple recent infarcts scattered throughout the left cerebral hemisphere, consistent with recurrent embolic events. Given the patient’s ongoing symptoms and imaging findings, an urgent CEA with primary closure was performed. The postoperative course was uneventful, and no further neurological events occurred (Figure 2).

### 3.5. Case 5

**Figure 3:**
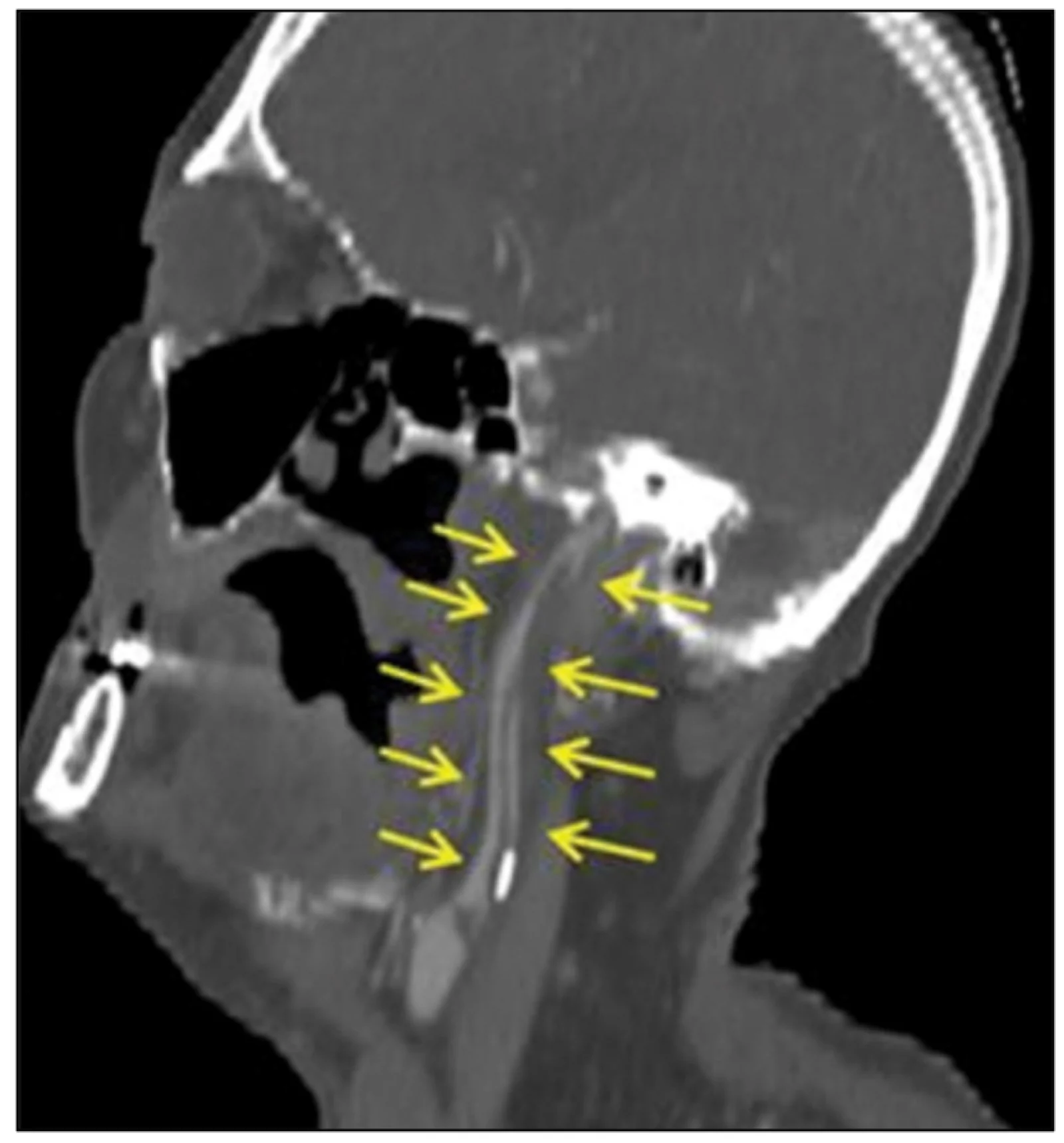
Sagittal computed tomography angiography (CTA) image demonstrating a carotid free-floating thrombus (CFFT) (arrows) attached to the carotid bulb and extending distally to the carotid siphon.

A 55-year-old man with a recently diagnosed colon adenocarcinoma presented with left-sided hemiparesis and confusion. Brain MRI demonstrated multiple acute cerebral infarcts. Color duplex ultrasonography revealed a <50% stenosis at the level of the right carotid bulb, as well as thrombosis of the right internal carotid artery (ICA). CTA further demonstrated a free-floating thrombus arising from an atherosclerotic plaque at the carotid bifurcation and extending distally to the carotid siphon, with a total length of approximately 7 cm (Figure 3).

**Figure 4:**
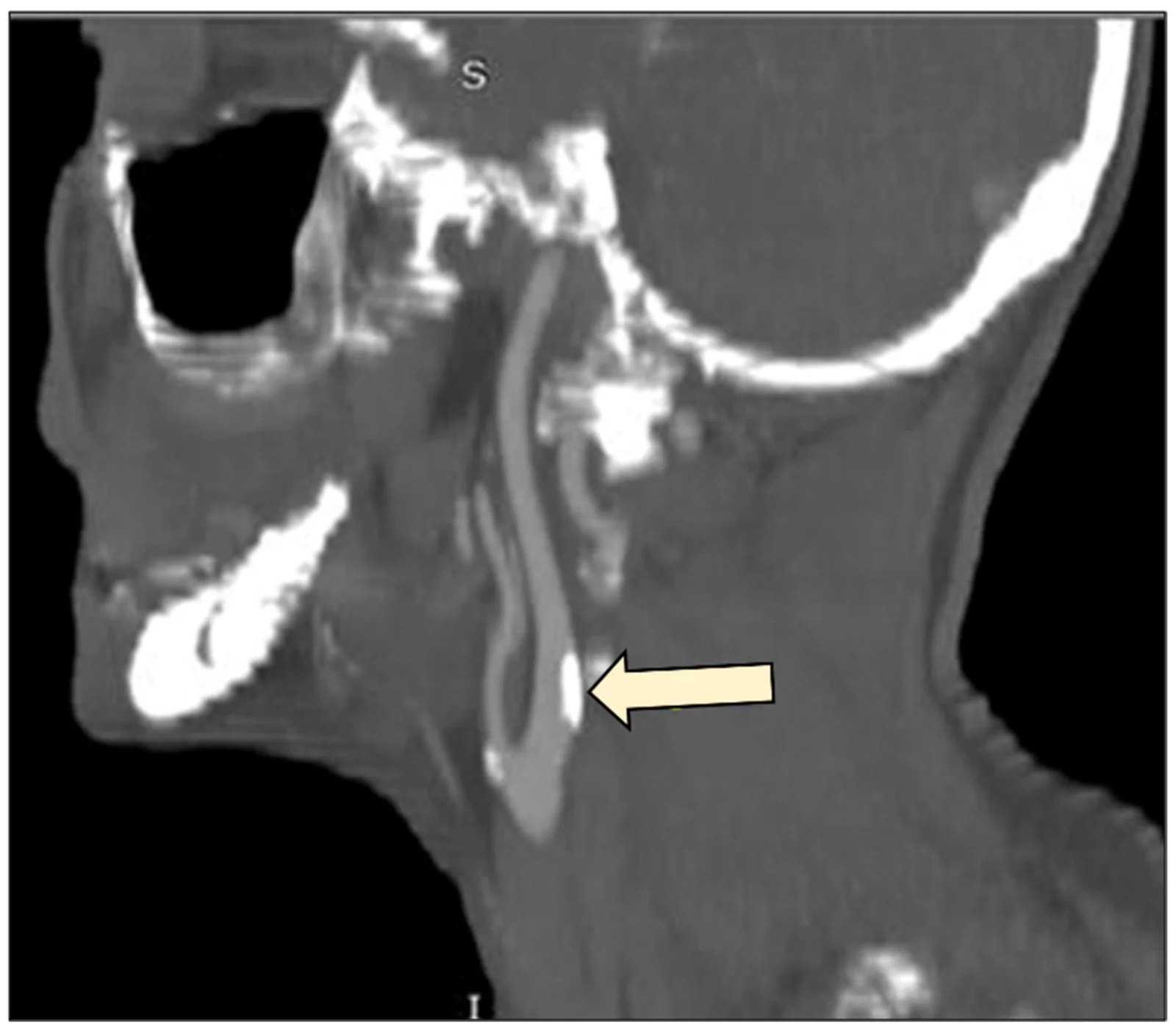
Total lysis of thrombus after treatment uncovers the responsible atheromatic plaque (arrows).

The patient was treated with enoxaparin 60 mg twice daily and acetylsalicylic acid 100 mg once daily. Gradual neurological improvement was observed, although mild residual weakness of the left upper extremity persisted. Follow-up CTA performed two weeks later demonstrated complete resolution of the thrombus, leaving only the underlying atherosclerotic plaque visible (Figure 4).

Medical treatment was continued, and the patient subsequently underwent an uneventful subtotal colectomy two months later. Following surgery, long-term secondary prevention was maintained with dual antiplatelet therapy.

## 4. Discussion

### 4.1. Epidemiology–Natural History

Most patients with CFFT are symptomatic (>90%), presenting with TIAs or ischemic stroke [1]. The clinical significance of CFFT lies in its potential for recurrent cerebral embolization through thrombus fragmentation. Less commonly, thrombus propagation may result in carotid artery occlusion. Nevertheless, the natural history is variable, ranging from thrombus stabilization to partial or complete spontaneous lysis, particularly under medical treatment [5]. Asymptomatic cases are uncommon and are usually identified incidentally during carotid duplex screening.

A male predominance has consistently been reported, with an approximate male-to-female ratio of 2:1 [1]. In more than 75% of cases, an underlying atherosclerotic plaque is considered the primary substrate for thrombus formation [6]. Hypercoagulable states are also frequently encountered, being present in nearly half of patients, and may be related to thrombophilia, pregnancy, inflammatory disorders, infection, or malignancy. Less common predisposing factors include carotid web, arterial dissection, fibromuscular dysplasia, and cardioembolic disease [6].

CFFT most commonly arises at the carotid bifurcation and may extend distally into the ICA. In a recent series, thrombi were located in the extracranial ICA in 62% of cases, the carotid bulb in 22%, the intracranial ICA in 14%, and the CCA in only 2% [7]. Within the same cohort, atherosclerosis was identified as the underlying etiology in 46% of patients, followed by carotid web or dissection in 20%, hypercoagulable states in 16%, and cardiac arrhythmias in 10% [7].

The risk of recurrent neurological events remains a major concern. In their systematic review and meta-analysis, Fridman et al. evaluated 58 case series and 83 case reports comprising 525 patients, of whom 345 underwent either medical or interventional treatment [6]. The combined 30-day risk of death, TIA, stroke, or silent ischemia on MΡΙ reached 17.1%, while the risk of stroke or death alone was 11.1%, highlighting the embolic potential of this condition. However, the available data were heterogeneous and largely retrospective. In a Cox regression analysis, neither anticoagulation versus no anticoagulation (HR 1.21; 95% CI 0.35–4.23; *p* = 0.76) nor early (<3 days) versus delayed (>3 days) intervention (HR 0.78; 95% CI 0.24–2.57; *p* = 0.69) was associated with a significant difference in the risk of silent ischemia, TIA, stroke, or death at 30 days [6].

Among patients for whom timing of intervention was reported, 94 underwent early CEA within the first three days after symptom onset, including 81 treated on the first day, whereas 64 underwent delayed intervention after three days, with a median time to surgery of 15 days (IQR 9–30 days) [6]. These findings illustrate both the absence of a standardized treatment strategy and the ongoing uncertainty regarding the optimal timing of intervention.

### 4.2. Histopathology of CFFT

The histopathological composition of a thrombus is not static but changes progressively over time. Shortly after formation, the thrombus consists mainly of fibrin, platelets, erythrocytes, and inflammatory cells. Classically, thrombus propagation differs between the arterial and venous circulation. Arterial thrombi are often described as propagating retrogradely, opposite to the direction of blood flow, whereas venous thrombi typically extend in the direction of flow toward the heart [8]. However, in free-floating carotid thrombi, the observed intraluminal morphology may not necessarily reflect the original direction of thrombus propagation, as the mobile distal component is influenced by local hemodynamic forces and blood flow patterns. During the early phase, it remains relatively soft and friable, which may explain both its embolic potential and its responsiveness to anticoagulation or thrombolytic therapies.

As the thrombus ages, the progressive deposition and cross-linking of fibrin, results in increased resistance of the thrombus to thrombolysis. Older thrombi undergo a gradual process of organization. Endothelial cells, fibroblasts and smooth muscle cells migrate into the thrombus, begin producing extracellular matrix and, eventually, form capillaries. Collagen deposition progressively increases, transforming the initially soft thrombus into a more compact and stable structure. In later stages, the lesion may become largely fibrotic and may even develop areas of neovascularization or recanalization [9].

These histological changes are likely to have important therapeutic implications. Fresh thrombi, which contain a higher proportion of fibrin and cellular elements, appear more susceptible to medical treatment and mechanical aspiration. In contrast, organized thrombi tend to be firmer and less responsive to pharmacological dissolution. This observation may partially explain the variability in treatment outcomes reported in the literature. Furthermore, thrombus maturation may influence the safety of intervention, as fresh thrombi are more prone to fragmentation and distal embolization during surgical or endovascular manipulation. A better understanding of thrombus age and composition could therefore contribute to more individualized treatment strategies in patients with CFFT [9].

### 4.3. Diagnosis

The diagnosis of CFFT can be established using color duplex ultrasonography (CDU), computed tomography angiography (CTA), magnetic resonance imaging/angiography (MRI/MRA), or digital subtraction angiography (DSA). In current clinical practice, treatment decisions are primarily based on the patient’s neurological status, CTA findings, and the evolution of the thrombus under medical therapy. Nevertheless, emerging imaging modalities, particularly vessel wall MRI and optical coherence tomography (OCT), may provide additional information regarding thrombus composition, plaque vulnerability, and thrombus maturation, potentially supporting more individualized treatment strategies in the future.

#### 4.3.1. Computed Tomography Angiography

CTA is currently considered the imaging modality of choice because of its wide availability, rapid acquisition, and excellent visualization of both the arterial lumen and the thrombus. Two characteristic imaging features have been described, the “donut sign” on axial images and the “finger sign” on sagittal reconstructions, both of which facilitate recognition of CFFT [10,11,12].

#### 4.3.2. Duplex Ultrasonography

Duplex ultrasonography remains an important first-line imaging modality because it is readily available, non-invasive, and allows rapid assessment of carotid stenosis and blood flow. However, its diagnostic sensitivity for CFFT is lower than that of CTA, with reported detection rates of approximately 53% in some series [13]. In selected patients, the “snake-fang sign” may be observed and has been associated with an increased risk of cerebral embolization [14].

#### 4.3.3. Digital Subtraction Angiography

Digital subtraction angiography can accurately demonstrate the presence of CFFT but is invasive and may not reliably distinguish the thrombus from the underlying atherosclerotic plaque [15,16]. Consequently, it is generally reserved for selected cases or performed in conjunction with endovascular intervention.

#### 4.3.4. Magnetic Resonance Imaging and Magnetic Resonance Angiography

MRI and MRA have traditionally been used in selected patients, particularly when radiation exposure or iodinated contrast administration should be avoided. Beyond anatomical assessment, recent advances in high-resolution vessel wall MRI have generated interest in the non-invasive characterization of thrombus biology and plaque vulnerability.

Experimental data suggest that fresh thrombi, owing to their higher erythrocyte and water content, may demonstrate imaging characteristics different from those of more organized thrombi, which typically exhibit features reflecting progressive fibrin organization, collagen deposition, and fibrosis. Although these observations have not yet been validated specifically in patients with CFFT, they support the concept that vessel wall MRI may eventually contribute to the assessment of thrombus maturation and treatment responsiveness [2,12].

From a clinical perspective, brain MRI currently has the greatest immediate impact on management. Detection of a large acute cerebral infarction or hemorrhagic transformation may influence both the timing of carotid revascularization and the use of anticoagulation because of the increased risk of reperfusion injury and intracranial hemorrhage [3,17]. In contrast, vessel wall MRI may identify vulnerable plaque features, including intraplaque hemorrhage and fibrous cap rupture, thereby supporting an underlying atherosclerotic source of embolism [18,19,20,21]. Although these findings are not currently incorporated into guideline-directed treatment algorithms, they may assist clinical decision-making in selected patients with recurrent embolic events or non-severe carotid stenosis.

#### 4.3.5. Intravascular Ultrasound and Optical Coherence Tomography

Emerging intravascular imaging techniques, including intravascular ultrasound (IVUS) and optical coherence tomography (OCT), provide high-resolution visualization of both the vessel wall and intraluminal pathology. Among these modalities, OCT offers superior spatial resolution by using near-infrared light rather than ultrasound.

OCT permits detailed assessment of plaque morphology, including plaque rupture, fibrous cap disruption, residual mural thrombus, and cholesterol-rich debris [15,16]. Preliminary studies also suggest that OCT may differentiate erythrocyte-rich from platelet-rich or mixed thrombi based on their characteristic optical backscattering properties, potentially explaining differences in responsiveness to anticoagulation. However, these observations are derived from limited observational studies and remain investigational.

Despite their promising diagnostic capabilities, the clinical role of IVUS and OCT in CFFT has not yet been established. Their invasive nature raises concerns regarding catheter manipulation within a vessel containing an unstable thrombus, with a theoretical risk of thrombus fragmentation and distal embolization. Furthermore, questions regarding safety, cost-effectiveness, and clinical utility remain unanswered. Consequently, these techniques should currently be regarded as adjunctive investigational tools rather than components of routine clinical practice [2].

Overall, CTA remains the cornerstone of CFFT diagnosis, while brain MRI has the greatest immediate influence on clinical management. Vessel wall MRI and OCT provide complementary information regarding thrombus maturation and plaque vulnerability that may eventually facilitate biology-guided treatment selection, although their role remains investigational and awaits prospective validation.

#### 4.3.6. Emerging Ultrasound Technologies

Recent advances in ultrasound technology have expanded the diagnostic capabilities of carotid imaging beyond conventional color duplex ultrasonography. Contrast-enhanced ultrasound (CEUS) improves visualization of plaque neovascularization, surface ulceration, and plaque morphology, while High Frame Rate Vector Flow Imaging (VFI) enables angle-independent assessment of complex flow patterns and local hemodynamic parameters such as wall shear stress. In addition, three-dimensional arterial ultrasound (3D-US) allows volumetric evaluation of carotid plaques and more comprehensive assessment of plaque geometry than conventional two-dimensional imaging [22,23].

Although these technologies have been primarily investigated in carotid atherosclerotic disease rather than carotid free-floating thrombus, they may eventually provide additional non-invasive information regarding plaque vulnerability, thrombus attachment, and local hemodynamics. Such complementary information could further refine patient selection and individualized treatment strategies. However, their application in CFFT remains investigational, and prospective studies are required before these techniques can be incorporated into routine clinical practice [24,25,26].

### 4.4. Treatment

The management of CFFT aims either to achieve thrombus resolution through antithrombotic therapy or to remove the thrombus using surgical or endovascular techniques [7]. Because of the rarity of the disease, no prospective randomized trials have directly compared medical and invasive treatment strategies, and current recommendations are therefore largely based on retrospective studies, case series, and expert opinion [5].

The 2023 ESVS Guidelines recommend initial anticoagulation with heparin (Class I recommendation), while invasive treatment is generally reserved for patients who experience recurrent neurological events despite medical therapy [3]. Intravenous thrombolysis is usually contraindicated. However, the recommendation supporting invasive treatment remains weak (Class IIb), reflecting the limited quality of the available evidence and the ongoing uncertainty regarding both the optimal treatment modality and the timing of intervention [3,5].

For the purposes of this review, failure of medical therapy refers primarily to recurrent or progressive neurological symptoms despite adequate antithrombotic treatment. Persistent thrombus, incomplete thrombus regression, or significant residual carotid stenosis on follow-up imaging may also support consideration of invasive treatment, particularly when accompanied by recurrent embolic events.

Treatment decisions should be individualized according to stroke severity, hemorrhagic risk, thrombus location, underlying etiology, and the availability of local expertise [5]. In patients with coexisting hypercoagulable conditions, anticoagulation appears to be the most rational first-line strategy [3]. In contrast, when an underlying atherosclerotic lesion is present, CEA or carotid artery stenting (CAS) may ultimately be required, particularly if significant residual stenosis remains after thrombus resolution [3,5]. Recurrent TIA or stroke despite optimal medical treatment is generally considered an indication for invasive intervention [3].

Current evidence supports initial anticoagulation in most patients; however, the optimal timing and type of invasive intervention remain individualized. While delayed procedures may be associated with a lower risk of thrombus fragmentation due to progressive thrombus organization, postponing intervention may also expose patients to recurrent embolic events [7]. The balance between these competing risks remains poorly defined. Stroke severity, the extent of cerebral infarction, and thrombus accessibility are additional factors influencing treatment selection [3]. From a surgical perspective, thrombi extending toward the skull base may be difficult to access safely and may require adjunctive techniques such as Fogarty catheter thrombectomy [27]. Endovascular thromboaspiration offers an alternative approach, particularly in the acute setting, but requires specialized expertise and equipment that are generally available only in experienced centers [28].

Recently, increasing attention has been directed toward the role of thrombus composition in determining treatment response. In their systematic review, Aouam et al. highlighted the potential importance of thrombus characterization in guiding the management of CFFT [29]. Traditionally, thrombi have been classified as red (erythrocyte-rich) or white (platelet and fibrin-rich), although this distinction is somewhat simplified. In reality, all thrombi are composed of fibrin, platelets, erythrocytes, and inflammatory cells, with differences arising mainly from the relative proportion of these components and the hemodynamic conditions under which the thrombus develops [30,31].

Although red thrombi are classically associated with venous thrombosis and low-flow states, they may also develop within the arterial circulation following rupture of an atherosclerotic plaque [31,32]. Studies of intracoronary thrombi have shown that erythrocyte-rich thrombi may be more prone to distal embolization during percutaneous coronary intervention than thrombi with a lower erythrocyte content [30]. Similarly, analyses of carotid endarterectomy specimens have demonstrated a predominance of red thrombi, which often exhibit features of plaque instability and may be associated with a higher risk of cerebrovascular events [33]. In contrast, thrombi occurring in patients with malignancy tend to be more platelet-rich and may exhibit characteristics of white thrombi [30,34].

OCT offers the possibility of characterizing the thrombus composition in vivo. According to Aouam et al., CFFT may display a distinctive “partial solar eclipse” appearance, in which the thrombus partially obscures the arterial lumen [18]. Red thrombi typically produce marked backscattering with signal attenuation due to their high erythrocyte content, whereas white thrombi generate a more signal-rich appearance reflecting a greater proportion of platelets and fibrin [29,32]. Such observations raise the possibility that thrombus composition may influence responsiveness to anticoagulant or antiplatelet therapy and perhaps even the risk associated with invasive intervention.

At present, however, OCT is not part of routine carotid imaging and its use remains limited by cost, availability, and concerns regarding catheter manipulation within a vessel containing a potentially unstable thrombus [29]. Nevertheless, the concept of tailoring treatment according to thrombus biology is intriguing and may represent an important step toward a more individualized approach to CFFT management. Future studies are needed to determine whether thrombus characterization can reliably guide therapeutic decision-making and improve clinical outcomes. The principal advantages, limitations, and potential indications of currently available treatment modalities are summarized in Table 2.

### 4.5. Antithrombotic Therapy

Traditionally, anticoagulation has been considered an effective strategy for thrombus resolution in most patients with symptomatic CFFT [1,35]. In their classic study published in 2007, Bhatti et al. reported thrombus lysis in 86% of patients treated with anticoagulation, while only 3% experienced neurological deterioration within 30 days [1]. More recently, Onalan et al. reported even higher rates of thrombus resolution, approaching 96% after two weeks of treatment [36].

It is generally accepted that anticoagulation is most effective in fresh thrombi. During the acute phase, particularly within the first week after thrombus formation, the clot remains rich in fibrin and cellular elements and is therefore more susceptible to lysis. As thrombus organization progresses, especially beyond the second week, collagen deposition and fibrosis gradually transform the lesion into a more compact and resistant structure. Consequently, older thrombi may be less responsive to medical therapy. In this context, high-resolution MRI may prove useful for estimating thrombus age and guiding treatment decisions [12]. Current imaging modalities, including CDU, CTA, and MRA, allow monitoring of thrombus resolution during follow-up [37].

Several reports have demonstrated successful thrombus resolution with anticoagulant therapy. In our fifth patient, who presented with colon cancer and a CFFT extending to the skull base, complete thrombus lysis was documented after two weeks of treatment with enoxaparin [38]. Similarly, Pelz et al. reported complete thrombus resolution in seven of eight patients during follow-up [37], while Karapurkar et al. described complete lysis after three weeks of enoxaparin therapy [39]. El Harake et al. demonstrated complete thrombus resolution within one week in approximately half of medically treated patients and reported a recurrence rate of 7% (3/39 patients) [7].

The optimal antithrombotic regimen remains uncertain. Available studies have not demonstrated significant differences between anticoagulant and antiplatelet therapy when used separately with regard to efficacy or safety outcomes [4,9,40,41]. Statin therapy should also be considered, as it has been associated with improved outcomes in patients with ischemic stroke [42]. Reported recurrence rates under medical treatment vary among studies. While some series have reported recurrence rates of approximately 14% [5], Aboul-Nour H. et al. observed a considerably lower rate of 1.4% at 30 days, and Torres et al. reported a recurrence rate of 2.5% [41,43]. Likewise, in the meta-analysis by Fridman et al., neither anticoagulation nor the absence of anticoagulation was associated with a significant difference in the risk of silent ischemia, TIA, stroke, or death at 30 days, although the authors acknowledged the limitations of the available data [6].

Despite these uncertainties, prompt initiation of anticoagulation with heparin is currently recommended by the 2023 ESVS Guidelines (Class I recommendation), reflecting the thrombotic nature of the disease process [3]. Some authors have suggested a subsequent transition to antiplatelet therapy once thrombus resolution has been achieved [44]. However, current guidelines do not specify the optimal agent, dose, or duration of treatment.

Future advances in thrombus characterization may allow a more individualized therapeutic approach. If thrombus composition could be reliably determined using techniques such as OCT, treatment might potentially be tailored according to the predominant thrombus subtype. As discussed previously, erythrocyte-rich (red) thrombi and platelet-rich (white) thrombi may exhibit different biological behaviors and treatment responses [16,32]. Although this concept remains speculative, a more personalized approach based on thrombus composition could improve recanalization rates and clinical outcomes.

Patients with stroke and CFFT who receive intravenous thrombolysis with rtPA should be closely monitored for signs of recurrent thromboembolism. Nevertheless, intravenous thrombolysis is currently not recommended by the ESVS Guidelines [3].

### 4.6. Carotid Thromboendarterectomy

Interventional treatment of CFFT may achieve higher rates of revascularization and potentially reduce stroke recurrence, although it is generally associated with higher rates of complications and mortality than medical therapy [4,40]. In selected patients who experience recurrent TIA or stroke despite optimal anticoagulation, and in whom the thrombus is surgically or endovascularly accessible, thrombectomy—either open or endovascular—may be considered, ideally following discussion within a multidisciplinary team [3]. Although the ESVS Guidelines advocate a relatively conservative approach, some authors have adopted a lower threshold for intervention [7].

El Harake et al. reported in 2023 on 50 patients with CFFT identified among 2038 stroke patients (2.45%), with a mean age of 58.2 years [7]. The principal indication for CEA was residual stenosis greater than 70% after medical treatment. Eleven patients (22%) underwent surgery, including eight with stenosis >70% and three with stenosis <70%, without any postoperative complications. The mean interval between diagnosis and operation was 12 ± 5.5 days. The authors attributed their favorable results, at least in part, to the delayed timing of surgery. In contrast, they suggested that the high recurrence rate reported in the series by Buchan et al. may have been related to early intervention, as surgery was performed after a mean of only 3 ± 0.3 days, with 11 of 16 patients undergoing operation within 27 h of diagnosis [33]. According to El Harake et al., surgical manipulation during the acute phase may increase the risk of thrombus fragmentation and distal embolization because of the friable nature of fresh thrombi [7].

Similar concerns have been raised by other authors. In the study by Bhatti et al., in which 82% of carotid endarterectomies were performed within the first seven days after diagnosis, neurological deterioration occurred in 9% of patients [1]. More recently, Khattab H. et al. reported effective thrombus resolution with antithrombotic therapy in seven of eight patients [10]. At three months, four patients demonstrated complete thrombus lysis and three partial lysis. Only one patient required CEA because of recurrent stroke on the 20th day after the index event. Based on these findings, the authors suggested that CEA should be considered not only after stroke recurrence, as recommended by the 2023 ESVS Guidelines, but also in patients with unsatisfactory thrombus resolution under medical treatment [10]. This strategy is largely consistent with the approach proposed by El Harake et al. [7].

Despite these recommendations, some groups continue to advocate early surgical intervention in carefully selected patients and have reported favorable outcomes. This was also the case in our third patient, who underwent early CEA without complications. Several case reports and small case series published after the 2023 ESVS Guidelines have described successful acute-phase surgery [3]. Shiozaki E. reported a successful CEA on the second day after presentation in a 42-year-old man who developed mild neurological deterioration despite medical treatment [45]. Similarly, Xu W. et al. performed CEA two days after the index stroke with good results [46]. Pensato et al. described urgent CEA in a young patient with COVID-19 pneumonia and severe hypercoagulability, again with a favorable outcome [47]. Interestingly, in that case no significant atherosclerotic disease was present, highlighting that urgent surgery may occasionally be considered even in patients whose primary pathology would otherwise favor anticoagulation.

Thrombus extension into the carotid siphon is generally regarded as a relative contraindication to open surgery because safe distal control cannot be achieved and restoration of flow may increase the risk of distal embolization [2,28]. Nevertheless, challenging current recommendations, Aouam et al. recently argued in favor of immediate surgical intervention whenever technically feasible, reserving percutaneous thrombectomy or thromboaspiration for patients unsuitable for open surgery. In their systematic review, Aouam et al. included eleven retrospective studies comprising 179 patients, excluding studies with fewer than five patients [29]. CEA was associated with the most favorable short-term (<30 days) and long-term (>30 days) outcomes, with lower rates of stroke or death compared with antithrombotic therapy and endovascular treatment (short-term: 2.6% vs. 6.7% vs. 5.0%; long-term: 0% vs. 6.5% vs. 0%). Antithrombotic therapy alone was associated with the highest rates of stroke or death during both follow-up periods. Based on these findings, the authors concluded that anticoagulation alone may be insufficient in many patients and that CEA could represent the preferred treatment option in those suitable for surgery [29].

However, these conclusions should be interpreted with caution. The systematic review by Aouam et al. is based exclusively on retrospective data and is therefore vulnerable to substantial selection bias. Additional limitations include the relatively small number of patients, the lack of standardized definitions of CFFT, and the omission of modern antithrombotic regimens from many of the included studies [28]. Furthermore, the timing of CEA was poorly reported, with detailed information available for only a small proportion of patients, among whom the mean interval to surgery was 14.6 days [29]. Finally, the strict inclusion criteria resulted in a cohort of only 179 patients, whereas the overall published experience with CFFT exceeds 500 patients and approaches 600 cases in some reviews [7]. Consequently, although the findings are thought-provoking, they are insufficient to support universal surgical intervention in all patients with CFFT.

### 4.7. Endovascular Techniques

Endovascular treatment represents an alternative to open surgery and includes thromboaspiration, stent retriever thrombectomy, and CAS. Although these techniques have been successfully applied in selected patients, robust evidence regarding their safety and efficacy remains limited [28,48,49]. In some cases, CAS has been used to trap the thrombus against the arterial wall and prevent further embolization [50].

Bhogal P. et al. reported in 2021 the use of Wallstent or CGuard stents in seven patients, with distal protection employed in all cases [50]. One patient experienced neurological deterioration, while another required implantation of three stents because of in-stent thrombus protrusion. Based on their experience, the authors suggested that intervention should be considered not only after recurrent symptoms despite medical therapy, but also in patients with severe flow-limiting stenosis caused by the thrombus [50].

Several authors have described successful thrombectomy using stent retrievers. Yamamoto Y. et al. reported favorable results in two patients treated with a stent retrieval technique under flow reversal protection [40]. In their approach, balloon occlusion of both the common carotid artery (CCA) and external carotid artery (ECA) was performed, resulting in reversal of flow within the internal carotid artery (ICA). A distal filter was additionally used for cerebral protection [51]. Similarly, Nagao Y. et al. utilized an Embotrap III stent retriever as a distal protection device [52]. Following balloon occlusion, thromboaspiration was performed with a syringe, and the residual atherosclerotic ICA stenosis was subsequently treated with Wallstent implantation [52].

Other groups have reported successful thromboaspiration techniques. Carr K. et al. described successful aspiration thrombectomy using a Penumbra device (NeuronMax, Penumbra™ (Alameda County, CA, USA)) in a 51-year-old woman presenting with symptomatic CFFT [28]. Likewise, Giragani S. et al. achieved complete thrombus removal in a 45-year-old man with crescendo TIAs despite anticoagulation, using a stent retriever in combination with distal filter protection [48].

Earlier reports also demonstrated the feasibility of carotid stenting in this setting. Park J.W. et al. deployed Wallstents in two patients with thrombi involving the CCA [53]. In another patient, a 50-year-old woman, successful thromboaspiration of a thrombus extending along the ICA and ECA was achieved after balloon occlusion of the CCA [53]. More recently, Lin et al. described a dual-protection carotid artery stenting (DPCAS) technique combining aspiration and stent retriever technology to minimize the risk of distal embolization during intervention [54].

Taken together, these reports demonstrate the technical feasibility of endovascular management in selected patients with CFFT. However, the available evidence is limited to case reports and small case series, and no clear superiority over medical treatment or carotid endarterectomy has been established. At present, endovascular treatment should be considered on an individual basis, particularly in patients who are poor surgical candidates or have thrombi that are difficult to access surgically.

### 4.8. Hybrid Techniques

Hybrid approaches have recently emerged as an alternative treatment option for selected patients with CFFT, combining the advantages of open surgical exposure with contemporary endovascular techniques [55]. The rationale behind these procedures is to minimize the risk of distal embolization while maintaining the ability to treat both the thrombus and the underlying carotid lesion during a single intervention.

One of the most widely discussed hybrid strategies is transcarotid artery revascularization (TCAR), which employs direct carotid access and cerebral protection through flow reversal. By reversing blood flow within the internal carotid artery during intervention, embolic material can theoretically be diverted away from the cerebral circulation, thereby reducing the risk of peri-procedural stroke [56,57]. This concept is particularly attractive in patients with CFFT, where manipulation of a friable thrombus may otherwise lead to distal embolization.

Christian Z.K. et al. reported in 2022 the successful treatment of a patient with CFFT using TCAR combined with aspiration thrombectomy and deployment of an ENROUTE carotid stent [55]. The procedure was performed after five days of dual antiplatelet therapy and resulted in complete thrombus exclusion without neurological complications. Similar to other endovascular approaches, TCAR offers the advantage of avoiding extensive surgical dissection while maintaining cerebral protection throughout the intervention.

Hybrid procedures may be particularly useful in patients who are considered high-risk candidates for conventional CEA, in those with anatomically challenging lesions, or in cases where thrombus morphology makes purely surgical or purely endovascular treatment less appealing [56,58]. Nevertheless, experience remains limited, and current evidence is restricted to isolated case reports and small series. As a result, the exact role of TCAR and other hybrid techniques in the management of CFFT has yet to be defined.

Future studies comparing hybrid, surgical, and purely endovascular approaches may help clarify patient selection criteria and determine whether cerebral flow-reversal systems provide a meaningful reduction in embolic complications during treatment of CFFT.

### 4.9. Timing of Intervention

In the absence of adequately powered prospective studies, the decision of whether and when to intervene with open, endovascular, or hybrid techniques remains one of the most challenging aspects of CFFT management. Although the current ESVS Guidelines recommend invasive treatment only after recurrent neurological events, this recommendation is based on a low level of evidence (Class IIb, Level C). Medical management achieves thrombus resolution in most patients, but failure rates of up to 15% at 30 days have been reported in several series [1,5,6]. More recent studies have reported lower rates, ranging from 1.6% to 7% [7,36,41,43]. Nevertheless, recurrent ischemic events continue to occur during medical treatment alone. 

Conversely, CEA has been associated with considerable perioperative stroke rates in some historical series [4,40,59,60]. For this reason, many authors advocate delayed intervention, arguing that thrombus organization reduces friability and lowers the risk of embolization during surgical manipulation. However, several case reports and small case series have demonstrated favorable outcomes even when surgery was performed during the acute phase. 

In a review of single-center retrospective studies, Vellimana et al. reported complication rates of up to 29% when CEA was performed within the first two weeks after presentation, whereas no complications were observed when surgery was delayed beyond two weeks, with the exception of the series by Buchan et al., in which one of six patients in the delayed-treatment group suffered a recurrent stroke [60]. On the other hand, Fridman S. et al. found no significant difference in the risk of TIA, stroke, death, or silent ischemia at 30 days when comparing interventions performed within three days versus after three days from symptom onset, although the available data were limited and heterogeneous [6]. 

A prolonged waiting period may also carry risks. Although a delay of two weeks allows further thrombus organization and stabilization, most recurrent neurological events appear to occur during the first days after diagnosis [5,6]. Müller et al. reported that no recurrent neurological events occurred beyond 11 days [5]. However, interpretation of these findings is difficult because 71% of patients in that series underwent intervention within the first nine days, and recurrence rates remained substantial in both treatment groups [5]. 

A reasonable compromise may be to consider CEA after the acute phase, approximately 5–7 days after symptom onset. By this time, progressive thrombus organization and collagen deposition may have reduced thrombus friability, potentially lowering the procedural risk. Repeat imaging with CDU, CTA, or MRA at this stage may also provide valuable information regarding thrombus resolution and the degree of residual stenosis. Patients with persistent severe stenosis or inadequate thrombus resolution could then be considered for surgical treatment. More delayed interventions, such as those performed after two or three weeks, may benefit from a more stable thrombus but at the cost of prolonged exposure to the risk of recurrent embolization during medical therapy. 

The experience reported by El Harake et al. is of particular interest in this regard. In their series, no postoperative strokes occurred following CEA, and the mean interval to surgery was 12 ± 5.5 days [7]. This suggests that intervention between the first and second week after the index event may represent a relatively safe therapeutic window. However, recurrent neurological events still occurred in 7% of medically treated patients (3/39), and it remains unknown whether some of these events could have been prevented by earlier intervention [7]. 

Despite these considerations, successful acute-phase CEA continues to be reported. While such cases demonstrate technical feasibility, the true procedural risk remains difficult to estimate because unsuccessful experiences are less likely to be published. We therefore believe that surgery during the very early phase carries an increased risk of thrombus fragmentation and distal embolization. At a minimum, intervention during the first 24 h should be approached with caution, as the thrombus is likely to be highly friable and immediate anticoagulation is generally warranted. 

An additional limitation of any time-based strategy is that the true age of the thrombus is usually unknown. Clinical presentation reflects the onset of symptoms rather than the onset of thrombus formation, and the lesion may have been present for days before becoming symptomatic. 

In contrast, endovascular techniques based on thromboaspiration may theoretically be most effective during the earliest stages of thrombus formation, when the thrombus remains soft and mechanically removable. To reduce the risk of distal embolization, contemporary procedures commonly employ distal protection devices, flow-reversal systems, or both. Stent-retriever thrombectomy represents another potential option in selected patients [27,51,61,62]. 

Taken together, the available evidence suggests that treatment timing should not rely solely on chronological presentation but also on thrombus characteristics, clinical stability, and underlying pathology. 

Ultimately, future treatment algorithms should move beyond a purely time-based approach and incorporate biological characteristics of the thrombus. High-resolution MRI may provide information regarding thrombus age and organization, while OCT may allow characterization of thrombus composition. Combining these parameters could facilitate more individualized treatment selection and help determine the optimal timing of intervention for each patient. The biological characteristics of a thrombus evolve over time and may influence its response to medical treatment, its susceptibility to embolization, and the risk associated with intervention. The potential impact of thrombus age and composition on treatment selection is summarized in Table 3. 

### 4.10. Thrombus Age and Composition as Determinants of Treatment Selection

Current treatment algorithms for CFFT are largely based on clinical presentation, thrombus location, and the occurrence of recurrent neurological events. However, these parameters provide limited information regarding the biological characteristics of the thrombus itself. Emerging evidence suggests that thrombus age and composition may represent equally important determinants of treatment response and procedural risk [12,29,32]. The following framework is intended as a conceptual synthesis of the preceding evidence and should be regarded as hypothesis-generating rather than a validated clinical decision algorithm.

Fresh thrombi are characterized by a higher content of fibrin, platelets, and erythrocytes and generally exhibit a softer and more friable structure. Such lesions may be more responsive to anticoagulation and mechanical aspiration, but they may also carry a higher risk of distal embolization during surgical or endovascular manipulation. In contrast, organized thrombi contain increasing amounts of collagen and extracellular matrix, resulting in a firmer and more stable lesion that may be less likely to fragment but also less likely to undergo complete lysis under medical treatment [8].

Similarly, thrombus composition may influence therapeutic efficacy. Erythrocyte-rich (“red”) thrombi may behave differently from platelet-rich (“white”) thrombi, reflecting differences in their underlying pathophysiology and biological properties [29,32]. Future advances in imaging, particularly high-resolution MRI and OCT, may allow non-invasive assessment of thrombus age and composition before treatment selection.

Based on these concepts, future treatment algorithms may evolve from a purely time-based approach toward a biology-guided strategy. Fresh, fibrin-rich thrombi without significant underlying carotid stenosis may be preferentially managed with anticoagulation or aspiration-based techniques, whereas organized thrombi associated with severe residual atherosclerotic disease may be more suitable for CEA or CAS. Although this concept remains hypothetical and requires prospective validation, it provides a potential framework for individualized management of patients with CFFT.

Several recent systematic reviews have comprehensively summarized the available evidence regarding the epidemiology, diagnosis, nomenclature, medical therapy, and interventional management of CFFT. Accordingly, the present review does not seek to duplicate these important contributions. Instead, our emphasis is on integrating these established data with emerging evidence regarding thrombus biology and advanced imaging in order to propose a conceptual framework for individualized treatment selection. Given the limited prospective evidence currently available, this framework should be regarded as hypothesis-generating and requires future validation in larger multicenter studies.

At present, only brain MRI findings demonstrating large cerebral infarction or hemorrhagic transformation have a direct impact on treatment timing and anticoagulation. In contrast, vessel wall MRI features (e.g., intraplaque hemorrhage or fibrous cap rupture) and OCT findings regarding thrombus composition or plaque morphology should currently be regarded as complementary investigational information that may support—but not determine—clinical decision-making.

**Figure 5:**
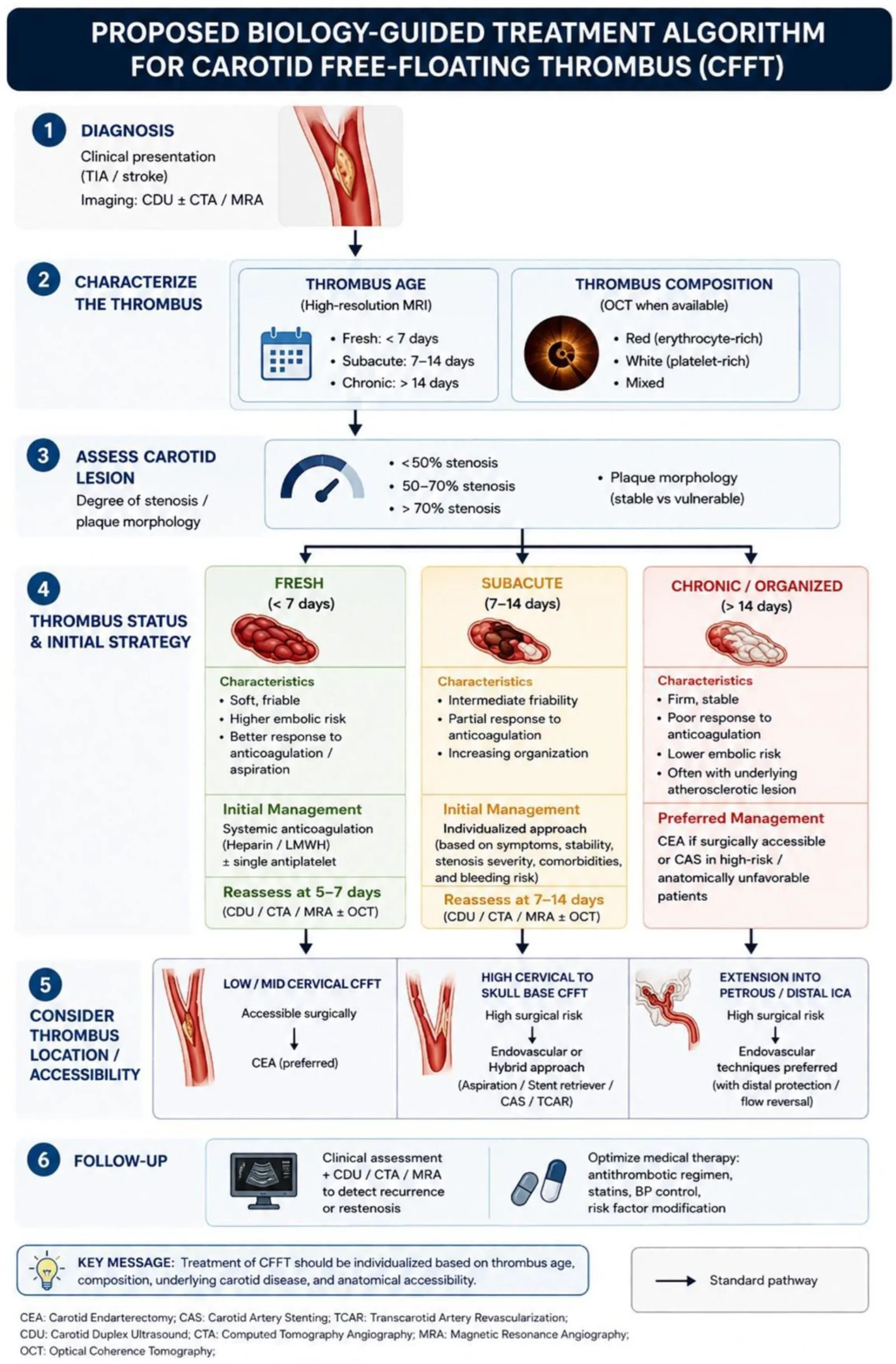
Proposed biology-guided treatment algorithm for carotid free-floating thrombus (CFFT). Conceptual framework illustrating a proposed biology-guided approach to treatment selection in carotid free-floating thrombus. The figure is intended to generate hypotheses and should not be interpreted as a validated clinical management algorithm. CEA, carotid endarterectomy; CAS, carotid artery stenting; TCAR, transcarotid artery revascularization; OCT, optical coherence tomography.

A proposed biology-guided treatment algorithm integrating thrombus age, thrombus composition, carotid lesion characteristics, and anatomical accessibility is presented in Figure 5.

## 5. Limitations

Several limitations should be acknowledged. First, the clinical component of this study is based on a small retrospective case series from a single institution, reflecting the rarity of CFFT. Consequently, no statistical comparisons can be performed and the findings should be interpreted as descriptive observations rather than definitive evidence. Second, the literature reviewed consists predominantly of retrospective studies, case reports, and small case series, which are inherently subject to selection bias, publication bias, and heterogeneity in patient populations, treatment strategies, and outcome reporting. As a result, direct comparisons between medical, surgical, and endovascular approaches remain difficult. Third, the proposed concepts regarding thrombus composition, thrombus maturation, and the potential role of advanced imaging modalities such as high-resolution MRI and OCT are largely based on emerging evidence and have not yet been validated in prospective studies specifically addressing CFFT. Moreover, a standardized etiologic workup was not performed uniformly in all patients because the cases were collected retrospectively over a 20-year period, during which diagnostic protocols evolved. Etiologic investigations were performed according to the individual clinical presentation and treating physician’s judgment. Furthermore, the absence of randomized controlled trials continues to limit the strength of current recommendations, particularly with regard to the optimal timing and selection of invasive interventions. Nevertheless, the combination of institutional experience and the contemporary literature provides a comprehensive overview of current management strategies and highlights important areas for future investigation. Finally, the proposed biology-guided treatment framework should be regarded as a conceptual, hypothesis-generating model rather than a validated clinical algorithm. It is based on the integration of the current literature, pathophysiological considerations, and the findings of a small retrospective single-center case series. Prospective multicenter studies incorporating standardized imaging protocols and longitudinal outcome data are required to validate this approach before it can be recommended for routine clinical practice.

## 6. Conclusions

Carotid free-floating thrombus remains a rare but potentially unstable vascular condition for which high-quality prospective evidence is still lacking. Initial anticoagulation continues to represent the cornerstone of management, while carotid endarterectomy, endovascular techniques, or hybrid procedures remain appropriate in carefully selected patients. Rather than proposing a new treatment algorithm, our review highlights the potential value of integrating thrombus biology, histopathological maturation, and advanced imaging characteristics into individualized clinical decision-making. This biology-guided framework should be regarded as a conceptual, hypothesis-generating model that complements existing management strategies and warrants validation in future prospective multicenter studies.

## Figures and Tables

**Table 1:** Clinical characteristics, management strategies, and outcomes of five patients with carotid free-floating thrombus (CFFT) treated at our institution. The table summarizes patient demographics, presenting symptoms, thrombus location, initial medical therapy, occurrence of recurrent neurological events, timing and type of surgical intervention, postoperative outcomes, and overall clinical course.

Case Number	Sex	Age	Symptoms	Site of FFT	Degree of Stenosis (%)	Thrombus Length (mm)	Medical Treatment	Initial Antithrombotic Regimen	Stroke Recurrence/ Timing	Open Surgical Treatment	Post-Operative Stroke	Outcome	Follow-Up Imaging
1	M	35	TIA (Numbness)	ICA	40	23	Initially	Aspirin + LMWH	Yes (1st day) Broca’s aphasia	Yes (1st day) urgent	No	Remained with Broca’s aphasia	Doppler Ultrasonography
2	M	40	Stroke/ Hemiparesis	ICA	30	18	Initially	Aspirin + LMWH	Yes (7th day) Hemiplegia	Yes (7th day) urgent	No	Remained with Hemiplegia	Doppler Ultrasonography + MRA
3	M	60	TIA	CCA	70	10	Initially	Aspirin + LMWH	No	Yes (3rd day) elective	No	Excellent	Doppler Ultrasonography
4 *	M	58	Crescendo TIA’s	ICA	80	16	Initially	Aspirin + LMWH	No	Yes (1th day) urgent	No	Excellent	Doppler Ultrasound
5 *	M	55	Mild stroke	ICA	<50	70	Main therapy	Aspirin + Enoxaparin	No	No	-	Total resolution of FFT after 15 days—Neurological improvement	Doppler Ultrasound + Angio-CT Scan

* Already published (REF 10, 27); TIA, transient ischemic attack; ICA, internal carotid artery; CCA, common carotid artery.

**Table 2:** Advantages and disadvantages of currently available treatment modalities for carotid free-floating thrombus (CFFT).

Treatment Modality	Main Advantages	Main Disadvantages	Potentially Suitable Patients
Anticoagulation	Non-invasive; widely available; high rates of thrombus resolution reported in several series; addresses underlying hypercoagulable states	Risk of recurrent embolization while awaiting thrombus resolution; incomplete lysis in a subset of patients; optimal agent and duration remain uncertain	Initial treatment in most patients; hypercoagulable states; extensive thrombi not amenable to intervention
Antiplatelet Therapy	Simple administration; favorable safety profile; useful for secondary prevention	Uncertain efficacy as sole therapy for CFFT; limited evidence regarding thrombus resolution	Adjunctive therapy; long-term secondary prevention; selected platelet-rich thrombi
Carotid Endarterectomy (CEA)	Simultaneous removal of thrombus and atherosclerotic plaque; definitive treatment of residual stenosis; extensive surgical experience	Risk of thrombus fragmentation and perioperative embolization; technically challenging in high cervical lesions; requires general or regional anesthesia	Recurrent neurological events; severe residual carotid stenosis; surgically accessible thrombi
Carotid Artery Stenting (CAS)	Less invasive than open surgery; avoids cervical dissection; treats residual stenosis	Potential embolization during lesion crossing; risk of in-stent thrombus protrusion; need for dual antiplatelet therapy	High surgical-risk patients; unfavorable neck anatomy; selected residual stenoses
Aspiration Thrombectomy	Immediate thrombus removal; potentially attractive for fresh thrombi; minimally invasive	Limited evidence; risk of distal embolization; requires specialized expertise and equipment	Acute-phase thrombi; thrombi extending beyond surgical accessibility
Stent Retriever Thrombectomy	Direct mechanical thrombus extraction; may be combined with protection devices	Limited experience in CFFT; procedural complexity; embolic risk during retrieval	Selected patients treated in specialized neurovascular centers
TCAR (Transcarotid Artery Revascularization)	Cerebral protection through flow reversal; combines surgical and endovascular advantages; avoids aortic arch manipulation	Limited clinical experience in CFFT; requires specialized equipment and training	High-risk surgical patients; selected anatomically suitable lesions
Hybrid Procedures	Allow combination of thrombectomy, cerebral protection, and treatment of underlying stenosis; individualized approach	Technically demanding; limited evidence; availability restricted to experienced centers	Complex lesions; patients unsuitable for isolated surgical or endovascular treatment
Intravenous Thrombolysis	Potentially rapid thrombus dissolution	Risk of thrombus fragmentation and recurrent embolization; currently not recommended by ESVS guidelines	No established role in routine CFFT management

Treatment selection should be individualized according to clinical presentation, thrombus characteristics, underlying etiology, anatomical accessibility, local expertise, and patient-specific risk factors.

**Table 3:** Potential impact of thrombus age and composition on treatment selection in carotid free-floating thrombus (CFFT).

Parameter	Fresh Thrombus (Acute Phase, <7 Days)	Subacute/Organizing Thrombus (7–14 Days)	Chronic/Organized Thrombus (>14 Days)
Dominant histological features	Fibrin-rich thrombus with abundant erythrocytes, platelets, and inflammatory cells	Progressive cellular lysis, macrophage infiltration, increasing collagen deposition	Predominantly fibrotic tissue with advanced organization and extracellular matrix formation
Mechanical properties	Soft, friable, highly mobile	Intermediate consistency with progressive stabilization	Firm, stable, less deformable
Risk of spontaneous embolization	Highest	Moderate	Lower
Risk of embolization during intervention	High due to thrombus friability	Intermediate	Lower
Expected response to anticoagulation	Highest likelihood of thrombus resolution	Variable response	Reduced response due to advanced organization
Expected response to thromboaspiration	Favorable	Potentially feasible	Less favorable
Suitability for intravenous thrombolysis	Theoretical benefit but currently not recommended by ESVS guidelines	Limited	Poor
Preferred imaging assessment	CTA, CDU, MRI for thrombus age estimation	CTA, CDU, MRI	CTA, CDU, MRI
Potential role of OCT	Characterization of thrombus composition (red, white, mixed)	Characterization of thrombus composition and organization	Assessment of residual plaque morphology
Residual carotid stenosis assessment	May be obscured by thrombus burden	Increasingly assessable during follow-up	Usually clearly defined
Potential role of medical therapy	Primary treatment strategy in most patients	Continued therapy if thrombus resolution is progressing	Less likely to achieve complete lysis
Potential role of carotid endarterectomy (CEA)	Technically feasible but potentially higher embolic risk	Potentially optimal balance between thrombus stabilization and recurrence prevention	Favorable surgical conditions if intervention remains indicated
Potential role of carotid artery stenting (CAS)	Selected cases with favorable anatomy and cerebral protection	Alternative to CEA in selected patients	Useful in surgically high-risk patients
Potential role of aspiration thrombectomy	Most attractive phase for aspiration-based techniques	Case-dependent	Limited role
Proposed treatment concept	Anticoagulation ± antiplatelet therapy; consider aspiration-based techniques in selected cases	Reassess thrombus resolution and residual stenosis; individualized decision regarding intervention	Consider definitive treatment of residual carotid pathology (CEA/CAS) if clinically indicated

This table represents a conceptual framework based on currently available evidence and expert opinion. Prospective studies validating treatment selection according to thrombus age and composition are currently lacking. The proposed timing categories represent a conceptual framework derived from current knowledge of thrombus biology and are not validated specifically for carotid free-floating thrombi.

## Data Availability

No new data were created or analyzed in this study.
